# Health services research in patients with breast cancer (CAMISS-prospective): study protocol for an observational prospective study

**DOI:** 10.1186/s12885-017-3926-9

**Published:** 2018-01-08

**Authors:** Susana García-Gutierrez, Miren Orive, Cristina Sarasqueta, Maria Jose Legarreta, Nerea Gonzalez, Maximino Redondo, Amado Rivero, Pedro Serrano-Aguilar, Xavier Castells, Jose Maria Quintana, Maria Sala, on behalf of REDISSEC-CaMISS group, Xavier Castells, Mercè Comas, Laia Domingo, Francesc Macià, Marta Roman, Anabel Romero, María Sala, Teresa Barata, Isabel Diez de la Lastra, Mariola de la Vega, Marisa Bare, Núria Torà, Joana Ferrer, Francesc Castanyer, Carmen Carmona, Susana García, Maximina Martín, Nerea Gonzalez, Miren Orive, Maria Amparo Valverde, Alberto Saez, Inma Barredo, Manuel de Toro, Josefa Ferreiro, Jose María Quintana, Jeanette Pérez, Amado Rivero, Cristina Valcárcel, María del Carmen Padilla, Maximino Redondo, Teresa Téllez, Irene Zarcos, Cristina Churruca, Amaia Perales, Javier Recio, Irune Ruiz, Cristina Sarasqueta, Jose María Urraca, Ma Jesús Michelena, Julio Moreno, Gaizka Mallabiabarrena, Patricia Cobos, Borja Otero, Javier Gorostiaga, Itsaso Troya

**Affiliations:** 1 Research Unit, Galdakao-Usansolo Hospital [Osakidetza] – Health Services Research on Chronic Patients Network [REDISSEC], Barrio Labeaga s/n, 48960 Galdakao, Bizkaia Spain; 2 Research Unit, Universitario Donostia Hospital – REDISSEC, BioDonostia Health Research Institute, Donostia, Gipuzkoa Spain; 3 Department of Applied Mathematics, University of the Basque Country, Leioa, Spain; 4 Department of Biochemistry, Costal del Sol Health Agency, Marbella, Spain; 5 Canary Foundation for Health Care Research (FUNCANIS) – REDISSEC, Madrid, Spain; 6 Evaluation Unit of the Canary Islands Health Service (SESCS), Tenerife, Spain; 7 Department of Epidemiology and Evaluation, Hospital del Mar Medical Research Institute (IMIM), Barcelona, Autonomous University of Barcelona-REDISSEC, Barcelona, Spain

**Keywords:** Breast cancer, Health services research, Clinical prediction rules

## Abstract

**Background:**

Though breast cancer remains a major health problem, there is a lack of information on health care provided to patients with this disease and associated costs. In addition, there is a need to update and validate risk stratification tools in Spain. Our purpose is to evaluate the health services provided for breast cancer in Spain, from screening and diagnosis to treatment and prognosis.

**Methods:**

Prospective cohort study involving 13 hospitals in Spain with a follow-up period of up to 5 years after diagnostic biopsy. Eligibility criteria: Patients diagnosed with breast cancer between April 2013 and May 2015 that have consented to participate in the study. Data collection: Data will be collected on the following: pre-intervention medical history, biological, clinical, and sociodemographic characteristics, mode of cancer detection, hospital admission, treatment, and outcomes up to 5 years after initial treatment. Questionnaires about quality of life (EuroQoL EQ-5D-5 L, the European Organization For Research And Treatment Of Cancer Core Quality Of Life Questionnaire EORTC QLQ-C30 join to the specific breast cancer module (QLQ-BR23), as well as Hospital Anxiety and Depression Scale were completed by the patients before the beginning of the initial treatment and at the end of follow-up period, 2 years later. The end-points of the study were changes in health-related quality of life, recurrence, complications and readmissions at 2 and 5 years after initial treatment. Statistical analysis: Descriptive statistics will be calculated and multivariate models will be used where appropriate to adjust for potential confounders. In order to create and validate a prediction model, split validation and bootstrapping will be performed. Cost analysis will be carried out from the perspective of a national health system.

**Discussion:**

The results of this coordinated project are expected to generate scientifically valid and clinically and socially important information to inform the decision-making of managers and the authorities responsible for ensuring equality in care processes as well in health outcomes. For clinicians, clinical prediction rules will be developed which are expected to serve as the basis for the development of software applications.

**Trial registration:**

NCT02439554. Date of registration: May 8, 2015 (retrospectively registered) .

## Background

Prevalence of breast cancer remains high worldwide [1]. Mortality rates have been decreasing since the 1970s. Screening programs and advences in adyuvant therapy have contributed to decrease mortality and this pathology has become in a chronic disease. l [2]. On the other hand, the development of new biomarkers and other diagnostic tools and new therapies could lead to a greater variability in clinical practice.

Several decision tools have already been created with the aim of predicting overall 5 or 10 year and disease-free survival [3–5]. In addition, with the increase in the life-expectancy in these women, it has become important to assess quality of life [6–8]. On the other hand, the course of breast cancer may be influenced by variables not directly related to the breast, such as other comorbidities and treatments [9–11].

This research was designed under the auspices of the Health Services Research on Chronic Patients Network (REDISSEC). This network was created to focus on three major issues: the challenge of managing the phenomenon of chronicity, the desire for more and better information, and a need to increase research capacity in the fields of health policies and services in Spain [12]. The overall objective of the CAMISS (abbreviation from the Spanish for health services research in breast cancer) research project is to evaluate the health services received by patients with breast cancer from screening, diagnosis and treatment to prognosis (complications, survival, and quality of life).

Sala et al. conducted the CAMISS-Retrospective study, which included 1086 women with breast cancer from a population-based screening program. These women were diagnosed with breast cancer between 2000 and 2008 and were followed-up to December 2013. The main objective of that study was to assess the impact of the mode of detection (screen-detected cancer vs. interval breast cancer) on overall survival and disease-free survival. Notably, however, symptomatic women were not included in this retrospective cohort and data were not collected on quality of life [13].

The CAMISS-Prospective study was designed by REDISSEC researchers in an effort to provide information on: 1) outcomes and their variability in breast cancer; 2) potential tools to improve the decision-making process from health system, professional and patient perspectives; and 3) the costs related to breast cancer in Spain. Our goal in this paper is to explain the design of the CAMISS-Prospective study, the second part of a comprehensive evaluation of health services research in patients with breast cancer in Spain. To our knowledge, this is the first research with this comprehensive perspective in Spain, considering not only clinical and economic outcomes but also in patient-reported outcomes. We will also combine retrospective data (from the CAMISS-Retrospective study) with this prospective research.

## Methods/Design

### Aim

The specific study objectives (which are set out in detail in Table 1) are, in brief: 1. to assess outcomes related to a) process of care (early diagnosis, access to health care and screening programs, delays in diagnosis, and variability in treatments), and b) patients (sociodemographic and clinical characteristics, including biomarkers, and patient-reported outcomes, such as quality of life and emotional state); 2. to create and validate prediction models (for changes in quality of life, relapse and death); and 3. to assess the costs associated with breast cancer care and its potential variations between Spanish regions.

**Table 1:** Objectives

1. Outcomes assessment	2. Creation of predictive models	3. Health services evaluation in breast cancer patients in Spain
To describe and analyse variability in outcomes by mode of detection, hospital, region and surgeon	• To create and validate predictive rules for relapse, mortality and complications	• To estimate the average costs of breast cancer care in Spain
To describe and analyse variability in the diagnostic process and treatment	• To identify risk factors for poor health-related quality of life 2 years after treatment	• To investigate differences between Spanish regions
To explore potential inequalities by age, education and socioeconomic level	• To validate our predictive rules for relapse, mortality and complications in a retrospective sample of patients from early detection programs	• To identify the most efficient process and treatments in breast cancer
To evaluate the impact of first hospital care (urgent or scheduled) and of delays on relapse, metastasis and death		

### Design and setting

CaMISS-Prospective is an observational analytic prospective cohort study. All the patients have been consecutively selected between April 15, 2013 and May 20, 2015, from 13 hospitals in 4 Spanish regions (Andalusia, Canary Islands, Catalonia, and the Basque Country). All participant centers belongs to the Spanish National Services were primary care and hospital emergency departments are free. Regions and participating hospitals are listed in Table 2.

**Table 2:** Patients recruited by area and hospital

Region	Hospital	Valid patients
Catalonia	Hospital del Mar, Barcelona	97
Basque Country	Hospital Galdakao-Usansolo, Bizkaia	197
	Hospital de Cruces, Bizkaia	246
	Hospital de Basurto, Bizkaia	125
	Hospital de Txagorritxu, Araba	229
	H.U. Donostia, San Sebastián, Gipuzkoa	245
	Instituto Oncológico, San Sebastian, Gipuzkoa	134
Andalusia	Hospital Costa del Sol, Malaga	80
	Antequera, Málaga	6
Canary Islands	Hospital Nuestra Sra de La Candelaria (Tenerife)	61
	Hospital Universitario de Canarias (Tenerife)	
	Complejo Materno-Insular (Gran Canaria)	36
	Clínica San Roque (Gran Canaria)	
Total		1456

### Study population

Women older than 18 years with an incident breast tumor will be included. The breast cancer diagnosis considered will be that reached after a biopsy of the tumor, including cases of ductal carcinoma in situ, invasive ductal carcinoma, tubular carcinoma, mucinous carcinoma, papillary carcinoma, cribriform carcinoma, invasive lobular carcinoma and lobular carcinoma in situ.

Symptomatic breast cancer will be included, as well as screened and interval breast cancer. The breast cancer screening program is public and universal in Spain. Following the recommendations of the European Guidelines for Quality Assurance in breast cancer screening and diagnosis [14], the Spanish Breast Cancer Screening Program provides free biennial screening to women who are between 50 and 69 years old. Recently, women aged from 45 to 49 years and 65 to 69 years are being incorporated into screening programs. Interval breast cancer is defined as primary breast cancer diagnosed in a woman who had a screening test, with or without further assessment, with a negative result, and diagnosed well before the next invitation to the screening test or before a period of time equal to the screening interval in a woman who has reached the upper age limit for screening [15].

Exclusion criteria are: diagnosis of sarcoma, lymphoma or inflammatory carcinoma; breast cancer recurrence; terminal illness; and a severe mental or physical condition or any other factor which interferes with the woman’s ability to complete the questionnaires. In addition, in situ carcinomas will be excluded from the survival analysis.

**Fig. 1:**
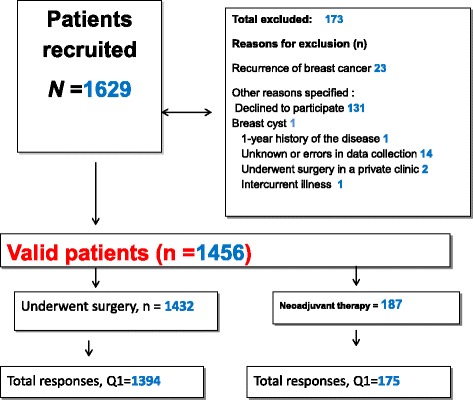
Flow chart

Socio-demographic and clinical data will be collected on women lost to follow-up. Figure 1 represents flow-chart of the recruitment and Table 2 represents data collection in participant centers.

### Information and data collection

Eligible patients are to be selected from the surgery lists if surgery is indicated or, when neoadjuvant therapy is the first treatment given, from the lists for oncological treatment. Patients are contacted by phone and informed of the study objectives and, if they agree to participate, asked to provide written informed consent.

**Fig. 2:**
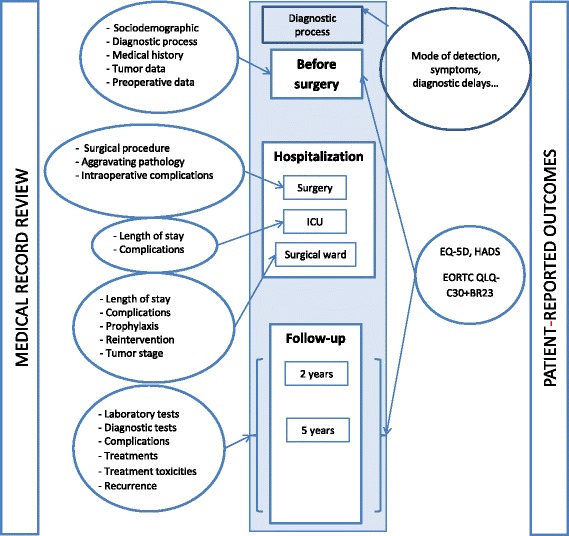
Data Collection process

Our goal is to follow-up participants for 5 years from the confirmation biopsy. Figure 2 summarizes the data collection process. Clinical and personal data will retrieved from medical records by trained reviewers before admission, and at 2 and 5 years after diagnosis. Information on hospital characteristics has been provided by the management of each hospital. In order to collect data on health-related quality of life, patients will be contacted to complete questionnaires administered by trained interviewers after their first treatment and at 2 years. The first interview is to be performed in the period between diagnosis and the date of surgery or the beginning of the neoadjuvant therapy in the cases in which this therapy is the first option. At 2 years, patient-reported outcomes will be self-reported by mail or through self-administered questionnaires completed during a follow-up visit. To increase the response rate up to three reminders will be sent: at 2 weeks and at 2 months after the first contact. In the interval, non-responders will be also telephoned to remind them that a questionnaire has been sent and also to offer them the option to respond over the phone if they prefer. If funding is available, the same procedure will be carried out at 5 years.

### Variables

Reviewers in each hospital were provided with a handbook with instructions to follow in the data collection process.

-Exposure variables:

- *Related to the patients’ personal background*: date of birth, sociodemographic variables (level of education, occupation of the patient or of the head of the household, marital status and living arrangements), height, weight, lifestyle habits, gynecological history (family history of gynecological cancer, oral contraceptives or hormonal replacement therapy, first and last menstruation date, menopausal status [pre- or postmenopausal], number of pregnancies and of births, breastfeeding [yes/no and duration]); and comorbidities (Charlson Comorbidity index) [16].
- *Related to the hospital*: number of beds, whether it is a teaching hospital, catchment population size, number of patients treated for breast cancer annually, and whether it has a breast cancer unit and medical or radiation oncology services.
- *Related to the process of care*: date of first contact with medical care (scheduled/emergency), time between the first symptoms and the first contact with medical services, time between the first medical appointment and histological diagnosis, time between diagnosis and treatment, and date of diagnosis.
- *Related to the pre-intervention tumor history*: mode of detection (symptomatic, screen-detected), symptoms (lump, lymph nodule, swelling or hardening of a part of breast, change in the size or shape of the breast, skin retraction, ulceration/wound, pain, secretion, inflammation, nipple retraction), date of the first symptom, clinical TNM classification (cTNM), additional diagnostic tests (ultrasound scan, magnetic resonance imaging [MRI], biopsy, computerized axial tomography [CAT], bone scan, galactography, ultrasound), histological type (infiltrating carcinoma, infiltrating ductal carcinoma, infiltrating lobular carcinoma, mucinous carcinoma, metaplastic carcinoma, intraductal carcinoma, and others) and serum marker levels (CA15-3, CA27.9, CEA, CA125).
- *Related to neoadjuvant treatment:* chemotherapy (regimen and whether it is completed), radiotherapy, hormone treatment, anti-HER2, and other therapies; and clinical and radiological response (assessed by MRI) categorized as no response (no changes or progression), weak partial response (if the tumor size reduced less than 50%), strong partial response (if the tumor size reduced by 50% or more), and complete response (no residual tumor) [17].
- *Related to surgery*: date, duration of the intervention, emergency vs scheduled, surgical technique (quadrantectomy, lumpectomy, segmentectomy, simple mastectomy, radical mastectomy, modified radical mastectomy, skin-sparing mastectomy, nipple-sparing mastectomy, contralateral prophylactic mastectomy, lymphadenectomy), time between diagnosis and intervention and/or pre-surgical adjuvant treatment, and intraoperative complications (bleeding, nerve injury, anesthetic complications, allergic reaction to the prophylactic antibiotic, others).
- *Related to anatomical pathology*: laterality, sentinel node biopsy (yes/no and results), histological type (intraductal, ductal, lobular, tubular, mucinous, medullary, cribriform, papillary, non-specific, others), degree of differentiation, pathological TNM (pTNM), location, size, distant metastases, vascular and nervous infiltration, number of lymph nodes analyzed and number positive, margin involvement, estrogen receptor/progesterone receptor status, Ki-67, P53, CK5/6, CK14, CK19, and HER2 expression, oncotype, and MammaPrint.
- *Related to admission*: setting (hospitalization vs. or ambulatory surgery), length of hospital stay in days after first intervention, in-hospital complications (seroma, wound infection, necrosis of skin flap, pneumothorax, brachial plexus pathology, and others), reintervention during admission, other medical treatments, and death.
- *Related to follow-up*: primary treatment: adjuvant postoperative treatment (radiotherapy, chemotherapy, hormone therapy, anti-HER2, and other therapy), date of treatments, reconstructive plastic surgery (yes/no, technique and date), other postoperative treatments (rehabilitation, psychologist/psychiatrist, others), and contact with social services; immediate complications and any reported during the follow-up period: chest wall and breast complications (seroma, post-surgery adhesions, soft tissue necrosis and recurrent skin infections), musculoskeletal (reduced arm mobility), lymphedema, neurological (paresthesias, neuropathy, cognitive dysfunction), pulmonary (pneumonitis, pulmonary fibrosis) cardiovascular morbidity (cardiomyopathy), psychological effects (anxiety), pain, other toxicities (ototoxicity, nephrotoxicity), reproductive health (premature menopause, infertility, sexual dysfunction), osteoporosis, weight gain, mycosis, and immunosuppression (agranulocytosis, lymphopenia); complications after reconstructive surgery (prosthesis infection, capsular contracture, others) and reinterventions, readmissions, death and their respective causes; and management of the disease during the follow-up period: diagnostic tests after surgery (CAT, positron emission tomography-CAT, biopsy, MRI, others), treatments (surgery, radiotherapy, chemotherapy, hormone therapy, anti-HER2, others), and number of follow-up visits per year (to the surgery/gynecology department, oncology department, rehabilitation unit, pain unit and palliative care unit).
*- Patient-reported measures*

The EORTC-QLQ-C30 (version 3.0) [18, 19] is an internationally validated health-related quality of life questionnaire that is widely used in cancer research. The core questionnaire is comprised of 30 items that assess five functioning domains (physical, role, cognitive, emotional, and social); eight cancer symptom domains (fatigue, pain, nausea and vomiting, dyspnea, insomnia, appetite loss, constipation, and diarrhea); financial difficulties, and global quality of life. The scores are transformed to a 0-100 scale, with a high score implying a high level of functioning or global quality of life, while for the symptom domains, higher scores indicate greater symptom burden. In conjunction with this core questionnaire (QLQ-C30), the breast cancer specific module, EORTC-QLQ-BR23, will be used [20]. This consists of 23 items assessing disease symptoms, side effects of treatment (surgery, chemotherapy, radiotherapy and hormonal treatment), body image, sexual functioning, and future perspectives. The scoring approach is identical to that for the QLQ-C30.

The self-complete version of the EuroQol generic health-related quality of life questionnaire (EQ-5D) [21] consists of two parts: the EQ-5D-5 L descriptive system and the EQ Visual Analogue scale (EQ VAS). The descriptive system comprises five dimensions (mobility, self-care, usual activities, pain/discomfort and anxiety/depression). Each dimension has five answer options that define different levels of severity. The EQ VAS records respondent’s self-rated health on a 20-mm vertical, visual analogue scale, ranging from 0 (worst imaginable health state) to 100 (best imaginable health state).

The HADS [22, 23] is a 14-item measure that evaluates psychological status. Seven items evaluate depression (the HADS-D subscale) and seven evaluate anxiety (the HADS-A subscale). A subscale score of 0 to 7 indicates the absence of anxiety or depression; 8 to10 a possible case of anxiety or depression; and 11 or higher a probable case of anxiety or depression.

2. Outcomes:
  - Objective outcomes: Second primary malignancies, complications, recurrence (local, regional, or remote), and death.
  - Patient-reported outcomes: Changes in the EORTC-QLQ-C30, EORTC-QLQ-BR23, HADS and EQ-5D-5 L scores between the time of inclusion in the study and the follow-up, initially at 2 years and then at 5 years.

### Safety and ethical considerations

We have obtained permission from the European Organization for Research and Treatment of Cancer to use the QoL questionnaires, EORT QLQ-C30 and QLQ-BR23 and from the EuroQoL Research Foundation to use the EQ-5D-5 L. We are using a version of HADS that has been validated by this research group [23].

Eligible patients will be informed verbally by trained research personal as well as in writing, and written informed consent will be obtained prior to enrollment. Patients may withdraw from the study at any time, during recruitment or follow-up, and all data collected will be treated as confidential. All participating hospitals have staff available to answer any questions that the patient or family members may have about the research.

Ethics Committe of each center approved the study. This study is registered with Clinical Trials.gov (identifier: NCT02439554).

### Follow-up

Regular follow-up visits will be performed up to 2 years at all 13 participating hospitals. In addition, a 5-year follow-up visit is also planned in all participating hospitals.

### Sample size calculation

We estimated the sample size based on the objective related to the creation and validation of a predictive model for which a relatively large sample size is required. The literature on prediction models indicates that a minimum of 10 outcome events are needed per predictor (relapse) [24]. Our aim is to include a limited but comprehensive list of variables (likely, not less than 10), in the multivariate regression models. Given this, we estimated that we needed at least 100 events of the dependent variable in the sample in order to ensure that the regression model converges adequately. It has been reported [25] that 7% of patients with breast cancer relapse within the first 2 years and considering this rate, we calculated the estimated sample size. Nevertheless, so far, based on 1456 patients, the relapse rate has been 4%, implying that no more than six variables should be included in the predictive models. We included all consecutive new cases until the sample size was achieved.

### Missing data assumptions and recoding of variables

Tumor definitions:

1. Bilateral breast cancer

Bilateral tumors with different pathological diagnoses at the time of diagnosis or up to 6 months later are described as synchronous bilateral breast cancer, while two breast tumors that occur in contralateral breasts at two different time points (more than 6 months of difference) are categorized as metachronous bilateral breast cancer. Lastly, two breast tumors with the same pathological diagnosis are considered bilateral metastatic breast cancer.

2) A recurrence or recurrent breast cancer is breast cancer that has come back during follow-up after a period in which cancer had not been detected. The cancer may come back in the same or opposite breast or chest wall. We recorded local, regional and metastatic recurrence.
3) A metastasis or metastatic breast cancer is defined as disease that has spread to distant sites of the body, such as the liver, lungs, bone, brain, and/or other tissues or organs [26].

### Symptomatology

Data will be collected on patient pre-intervention disease-specific symptoms.. A dichotomic variable was created based in the presence or absence of any symptom.

### Complications

A checklist (yes/no) is used for complications throughout the course of the follow-up period (intra-surgical, during hospital admission, and up to 2 and 5 years after the intervention, including complications related to reconstructive surgery). When there is no information on complications in the medical record, it will be assumed that none occurred.

Surgical severity: We record the use of the following surgical techniques (ranked from least to most complex): conservative surgery (tumorectomy, quadrantectomy, and segmentectomy), simple mastectomy, radical mastectomy, modified radical mastectomy, skin-sparing mastectomy, nipple-sparing mastectomy, areola-sparing mastectomy, breast reconstruction and contralateral prophylactic mastectomy.

pTNM: Staging is performed following the American Joint Committee on Cancer [27, 28], being pTNM considered except for cases who received neoadyuvant therapy (cTNM).When there are no pTNM staging data, the analogous cTNM will be used, otherwise, missing value will be recorded. In cases of bilateral cancer, we will consider the final stage as a peak between right and left breast. In the cases of Tx, Nx or Mx, we will consider the disease to be T0, N0 or M0. If cM is missing, then cM will be considered to be 0.

### Statistical analysis

- Descriptive statistics: Mean and standard deviations for continuous variables (or median and interquartile ranges, when the observed variables do not follow a normal distribution) and frequencies and percentages for qualitative variables.
-Bivariate analysis:The Student’s t-test or the non-parametric Wilcoxon test (for non-normal distributions) will be applied for two-level outcomes, and ANOVA analysis or a Kruskal–Wallis test (for non-normal distributions) where there are three or more categories in the outcome. Otherwise, for categorical variables, the Chi-square test (or Fisher’s Exact method, where required) will be used. Multivariate models will be used where appropriate to adjust for confounders.
-Creation and validation of predicitive models:. Participants will be randomly divided into two groups: the derivation (60%) and validation (40%) cohorts. The study unit will be the patient (each patient being included only once). The predictive model will be created with the derivation group (group 1). Initially, univariate analyses will be performed, to identify variables related to the selected outcomes. Variables with a *p* < 0.20 will be entered into a multivariate logistic regression model, when outcome variables are dichotomous (mortality, re-admissions or relapse, major complications). Statistically significant variables will be included in the final model. Based on its estimated contribution in the multivariate logistic regression model, a score will be assigned to each variable. From this, a severity risk score will be created with the receiver operating characteristic curve. One cut-off point will be selected, namely, that giving the best balance between sensitivity and specificity. For the continuous outcomes (changes in health-related quality of life), a general lineal model will be used. The validity of the model and the score will be tested in the validation sample (group 2) and also in the retrospective sample (group 3). We will calculate the sensitivity, specificity, and area under the receiver operating characteristic curve (AUC), and *p* values for comparisons of AUCs between the groups. The models will be calibrated using the Hosmer-Lemeshow test. A multilevel analysis will be performed with patients (level 1) nested within hospitals (level 2).

Cost analysis: The perspective of the cost analysis is that of a public hospital in a national healthcare system, and therefore, only direct medical costs (DMCs) will be included. DMCs will be derived from healthcare use registered in the medical records. Data on resource use will be obtained for the periods of 24 and 60 months after baseline, that is, since diagnosis. This includes data on medical visits; hospital admissions; laboratory tests, imaging studies, and other diagnostic procedures; and treatments including surgery, medication (chemotherapy and other) and radiotherapy. Little used and/or low cost resources will be not considered. The value of resources used by patients is to be calculated in terms of the relevant unit costs and the average cost per patient in the sample. The unit costs will be obtained for each resource from the accounting system of participating hospitals. Unit costs will be multiplied by the resource quantities to obtain the annual cost for each patient. All costs will be assigned in euros of the year in which the resource has been used; no discount rate will be needed due to the short time horizon.

Costs will be aggregated and classified according to the following categories: outpatient clinic visits (number of visits to specialists); hospitalization (total length of hospital admissions, length of stay in intensive care unit and day hospital visits); laboratory tests (laboratory tests performed through ambulatory care); and imaging studies (ultrasonography, axial computerized tomography, magnetic resonance, radiography and other imaging studies related to the diagnosis, and treatment and associated complications); diagnostic procedures (procedures related to the monitoring of the disease and associated complications); surgery (surgical procedures related to the treatment and associated complications); medication; radiotherapy.

### Quality assurance

The reviewers will be provided with a handbook designed by the main researchers together with clinical collaborators and receive specific instructions for the identification and collection of relevant data. During the study, they will be also supervised by the main researcher and clinical collaborators. Each reviewer of each participating hospital has an “ad hoc” database with a specific username and password, in which all the data are to be stored. Personal data that identify patients will be separated from the clinical data and patient-reported outcomes. Patient identification number will be used always for data management.. In addition, in each hospital there is a project manager helping reviewers, coordinating the study and ensuring that all processes comply with standards for good practice. Once a month, we will assess the quality of the data collection process..

### Duration of the project

The project is planned to last for at least 3 years divides in recruitment (1 year) and follow-up (2 years), At least 6 months will be required to finish error correction process and database cleaning. In a second stage, it is envisaged that patients will be followed-up at 5 years after their diagnostic biopsy (but, as mentioned above, this depends on funding).

### Project management

Coordination committee responsible for all decisions is comprised by study leaders. This study has five study leaders, from five research groups belonging to REDISSEC, who are responsible for each of the objectives. Dr. M Sala is responsible for the general coordination as well as the evaluation and development of predictive models related to survival and maintenance of remission in women with breast cancer participating in early detection programs (interval/screening cancers). She is coordinator of the CAMISS-Retrospective study. Dr. S García-Gutiérrez is responsible for the objective of creating and validating of prediction models. Dr. C Sarasqueta will be responsible of assess outcomes and to the influence of delays on outcomes to M Redondo. Lastly, objectives related to economic assessment will be pursued by L García Pérez.

## Discussion

Finally, 1629 patients have been recruited. The basic characteristics of the sample are summarized in Table 3, stratified by whether the women have undergone surgery.

**Table 3:** Basic description of the participating women

	*Total N = 1456*	
	*Surgery*	*No surgery*
	1432 (98.35%)	24 (1.65%)
Age, years^a^	57.604 (11.917)	80.575 (11.257)
Charlson comorbidity index^a^	0.325 (0.752)	0.958 (1.197)
Initial treatment		
Conservative	648 (45.25%)	–
Simple mastectomy	203 (14.18%)	–
Radical mastectomy	38 (2.65%)	–
Radical modified mastectomy	46 (3.21%)	–
Neoadjuvant therapy	165 (11.52%)	22 (91.67%)
Adjuvant therapy		
Chemotherapy^1^	455 (31.77%)	
ACT	166 (11.59%)	
TAC	80 (5.59%)	
CMF	8 (0.56%)	
FEC	42 (2.93%)	
FEC-Taxane	41 (2.86%)	
TC	35 (2.44%)	
Other	88 (6.15%)	
Radiotherapy (yes)	1126 (78.63%)	
Hormone therapy	1146 (80.03%)	
Tamoxifen	484 (33.8%)	
Tamoxifen +GnRH analogues^2^	50 (3.49%)	
Tamoxifen +Aromatase inhibitors	12 (0.84%)	
Aromatase inhibitors	639 (44.62%)	
Others	26 (1.82%)	
TNM^3^		
0	135 (9.43%)	0
I	683 (47.7%)	2 (8.33%)
II	453 (31.63%)	5 (20.83%)
III	134 (9.36%)	4 (16.67%)
IV	18 (1.26%)	11 (45.83%)

1.-Chemotherapy:

*ACT* Adriamycin/ doxorrubicine, cyclophosphamide + taxane (docetaxel / paclitaxel),

*TAC* docetaxel, Adriamycin, cyclophosphamide,

*CMF* cyclophosphamide, Methotrexate 5-Fluorouracil

*FAC* 5-fluorouracil, Adriamycin (Doxorubicin), cyclophosphamide- FEC: 5-fluorouracil, epirubicin, cyclophosphamide

*FEC-Taxane* FEC + paclitaxel

*TC* Taxane, cyclophosphamide

2.- *GnRH* Gonadotropin-releasing hormone analogues

*3.-pTNM* pathological tumor-node-metastasis staging in patients who underwent surgery

^a^Means and, in brackets, standard deviation

### Problems anticipated

Response rate and the difficulty of obtaining all the data required are the main problems of this study. To reduce the risks of low response rates and high losses to follow-up, a great effort is done to explain patients the objectives of the study in several times (at the enrollment and during the follow-up visits). Questionnaires will be sent Up to three ltimes by mail to patients and the option of completing the questionnaires over the phone is available upon request. In addition, regular contact will be maintained with all patients. To minimize the difficulties related to data retrieval from health records, all reviewers have received specific training, as well as a handbook to help them with the follow-up process.

### Expected outcomes of the study

The results of this coordinated project are expected to generate scientifically valid and clinically and socially important information to inform the decision-making of managers of screening programs, the authorities responsible for ensuring equality in the care process as well in health outcomes. For clinicians, clinical prediction rules will be developed which are expected to serve as the basis for software applications. Our intention is to create tools that will be easy to use, preferably to be added to electronic health records. This would allow physicians and patients themselves to consider the individual risk at the time of appointments, to guide their decisions. Such tools could also be used in evaluation of health services by health managers. Although here we describe in detail the protocol for 2 years of follow-up, our intention is to follow this cohort for longer (at least up to 5 years).

### Dissemination of results and publication policy

REDISSEC-CAMISS (Health Services Research in Breast Cancer) group has been established, For publication purposes, an author has to have contributed to each of the following activities: 1) conception/design and/or analysis/interpretation, 2) writing of the manuscript, and 3) approval of the final version, and take public responsibility for the content of the paper. All co-authors have to review and agree with the contents of the manuscript as submitted. Study and manuscripts will follow the STROBE guidelines for conducting and disseminating observational studies and the TRIPOD statement for reporting of a multivariable prediction model for individual prognosis or diagnosis [28]. The main study results will be disseminated in the media,. The main results of the project will also be linked to a website, created ad hoc for this project: http://www.CaMISS.info [29].

## Abbreviations

Anti-HER2: anti HER2 receptor treatment
CA125: Cancer Antigen 125
CA15-3: Cancer Antigen 15.3
CA27.9: Cancer Antigen 27.9
CAT: Computerized axial tomography
CEA: Carcinoembryonic Antigen
CK14: Cytokeratin 14
CK19: Cytokeratin 19
CK5/6: Cytokeratin 5/6
cTNM: clinical TNM
EORTC QLQ-BR23: European Organization For Research And Treatment Of Cancer specific breast cancer module
EORTC QLQ-C30: European Organization For Research And Treatment Of Cancer Core Quality Of Life Questionnaire
EQ VAS: EuroQol Visual Analogue scale
EQ-5D: EuroQol generic health-related quality of life questionnaire
EQ-5D-5 L: EuroQol descriptive system
HADS: Hospital Anxiety and Depression Scale
HER2: human epidermal growth factor receptor 2
Ki67: Antigen Ki67
Mammaprint: Amsterdam 70-gene profile
MRI: Magnetic Resonance Imaging
Oncotype: 21-gene recurrence score
P53: P53 gene
pTNM: pathological TNM
